# Lipid A from *Oligotropha carboxidovorans* Lipopolysaccharide That Contains Two Galacturonic Acid Residues in the Backbone and Malic Acid A Tertiary Acyl Substituent

**DOI:** 10.3390/ijms21217991

**Published:** 2020-10-27

**Authors:** Adam Choma, Katarzyna Zamłyńska, Andrzej Mazur, Anna Pastuszka, Zbigniew Kaczyński, Iwona Komaniecka

**Affiliations:** 1 Department of Genetics and Microbiology, Institute of Biological Sciences, Maria Curie-Sklodowska University, Akademicka 19, 20-033 Lublin, Poland; adam.choma@poczta.umcs.lublin.pl (A.C.); k.zamlynska@poczta.umcs.lublin.pl (K.Z.); mazur@hektor.umcs.lublin.pl (A.M.); ak.pastuszka@poczta.umcs.lublin.pl (A.P.); 2 Laboratory of Structural Biochemistry, Faculty of Chemistry, University of Gdansk, Wita Stwosza 63, 80-308 Gdansk, Poland; zbigniew.kaczynski@ug.edu.pl

**Keywords:** galacturonic acid, lipid A, lipopolysaccharide, malic acid, structure elucidation, VLCFA

## Abstract

The free-living Gram-negative bacterium *Oligotropha carboxidovorans* (formerly: *Pseudomonas carboxydovorans*), isolated from wastewater, is able to live in aerobic and, facultatively, in autotrophic conditions, utilizing carbon monoxide or hydrogen as a source of energy. The structure of *O. carboxidovorans* lipid A, a hydrophobic part of lipopolysaccharide, was studied using NMR spectroscopy and high-resolution mass spectrometry (MALDI-ToF MS) techniques. It was demonstrated that the lipid A backbone is composed of two d-Glc*p*N3N residues connected by a β-(1→6) glycosidic linkage, substituted by galacturonic acids (d-Gal*p*A) at C-1 and C-4’ positions. Both diaminosugars are symmetrically substituted by 3-hydroxy fatty acids (12:0(3-OH) and 18:0(3-OH)). Ester-linked secondary acyl residues (i.e., 18:0, and 26:0(25-OH) and a small amount of 28:0(27-OH)) are located in the distal part of lipid A. These very long-chain hydroxylated fatty acids (VLCFAs) were found to be almost totally esterified at the (ω-1)-OH position with malic acid. Similarities between the lipid A of *O. carboxidovorans* and *Mesorhizobium loti*, *Rhizobium leguminosarum*, *Caulobacter crescentus* as well as *Aquifex pyrophylus* were observed and discussed from the perspective of the genomic context of these bacteria.

## 1. Introduction

*Oligotropha carboxidovorans* strain OM5 is a Gram-negative slightly curved rod bacteria, possessing one lateral flagellum [1]. This bacterium was isolated from the soil of wastewater sewage treatment settling ponds near Götingen in Germany and was previously described as *Pseudomonas carboxidovorans* strain OM5 [2]. *O. carboxidovorans* can live in heterotrophic and chemolitho-autotrophic conditions. In the latter case, these bacteria can utilize carbon monoxide as a sole source of carbon and energy. Aerobically, they are able to oxidize approximately six molecules of CO to CO_2_ and simultaneously assimilate one of the produced CO_2_ molecules in the pentose phosphate pathway [3]. *Oligotropha* are economically important microorganisms, as they can oxidize syngas (i.e., a mixture of CO, CO_2_, and H_2_) generated by biogasification of organic wastes [4]. Globally, such types of bacteria remediate more than 100 million tons of carbon monoxide from the atmosphere [5]. Heterotrophically, in aerobic conditions, these bacteria are able to use fixed carbon components (mainly organic acids) as a source of carbon and energy [6,7]. *Oligotropha* species interact with other bacteria forming co-aggregates. This phenomenon is important for proper settling of the sludge [8].

16S rDNA sequencing showed that *O. carboxidovorans* is phylogenetically related to the members of *Bradyrhizobiaceae* family, i.e., *Bradyrhizobium* sp. BTAi1, *B. japonicum* USDA110, and *Nitrobacter hamburgiensis* X14 [6]. Although the genomes of all these strains share some common genes and operons, these bacteria differ in their metabolism, which allowed them to adapt to very diverse environments. A huge diversity in the lipid A structure can be observed within the group of soil borne bacteria, called rhizobia, and the structure of several rhizobial lipids A has been described in details over the last twenty years [9]. Some of them, e.g., *Sinorhizobium meliloti*, produce a classical enterobacterial lipid A model, containing a biphosphorylated glucosamine-based lipid A backbone. Some species, e.g., *Rhizobium leguminosarum*, modify their lipid A via oxidation of proximal glucosamine (Glc*p*N) to 2-aminogluconate (Glc*p*AN). There is also a group, e.g., all members of the *Mesorhizobium* genus and the majority of species belonging to the *Bradyrhizobium* genus, which possess lipid A built of 2,3-diamino-2,3-dideoxy-d-glucose (Glc*p*N3N) instead of Glc*p*N, substituted at position C-1 with uronic acid. Lipid A of *Mesorhizobium* is in addition partly phosphorylated at position C-4’ [10,11]. In turn, phosphate is absent in the lipid A of *Bradyrhizobium*, but position C-4’ is occupied by two mannose residues forming a pentasaccharidic lipid A backbone [12,13,14]. The lipid A backbone in all Gram-negative bacteria is acylated by 3-hydroxy fatty acids, which are amide-linked at position C-2 and C-2’of Glc*p*N and C-2, C-3, C-2’, and C-3’ of Glc*p*N3N and ester-linked at position C-3 and C-3’ of Glc*p*N. Almost all members of rhizobia have very long-chain hydroxylated fatty acids (VLCFAs) linked as secondary substituents of 3-hydroxy fatty acids. VLCFAs contain 26 to 34 carbon atoms in the acyl chain, but rhizobia usually esterify their lipids A with 27-hydroxyoctacosanoic acid, which can be further acylated by 3-hydroxybutyrate as a tertiary residue. The presence of at least two or three very long-chain hydroxylated fatty acid (VLCFA) chains furnishing lipid A as secondary acyl residues has been confirmed in *Bradyrhizobium* [13,14].

Almost 30 years ago, it was shown that, similar to the bradyrhizobia, *O. carboxidovorans* (*Pseudomonas carboxydovorans*) contain 2,3-diamino-2,3-dideoxy-d-glucose in their lipid A [15]. The aim of presented study was to complete the structural analysis of lipid A isolated from the *O. carboxidovorans* OM5 lipopolysaccharide using modern analytical techniques, such as NMR spectroscopy and high-resolution mass spectrometry. Additionally, since the full genome sequence of *O. carboxidovorans* OM5 has been published [6,16], the comparative analysis of the region responsible for lipid A biosynthesis and its fatty acid substitution was performed. The corresponding gene sequences described for *Mesorhizobium loti* as well as *Rhizobium leguminosarum* and *Sinorhizobium meliloti* strains were used as the reference.

## 2. Results

### 2.1. Isolation and Chemical Analysis of O. carboxidovorans Lipid A

**Figure 1:**
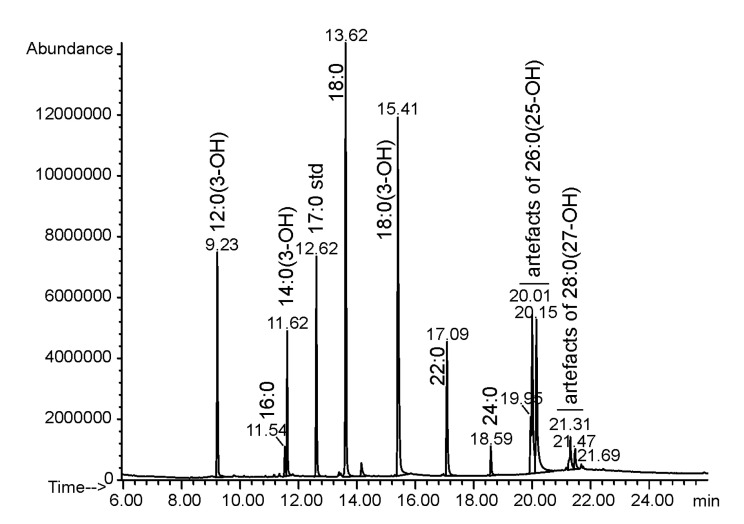
Fatty acid composition of *O. carboxidovorans* OM5 lipid A. Signals around 20 min and 21.30 min represent three isomers of the dehydrated form of 26:0(25-OH) and 28:0(27-OH), respectively, derived during strong acid hydrolysis (4 M HCl aq., 100 °C, 4 h). For conditions of gas chromatography, see Materials and Methods section. Artefacts mean hydrolytically dehydrated hydroxy fatty acids. Std—internal standard.

The LPS of *O. carboxidovorans* strain OM5 was extracted from delipidated and enzymatically digested cells with the hot 45% phenol/water method [17,18]. The LPS was found mainly in the water phase. Lipid A was obtained by mild acid (1% acetic acid) hydrolysis of LPS and was subjected to fatty acid and sugar analyses. The sugar components of the *O. carboxidovorans* lipid A backbone were represented by 2,3-diamino-2,3-dideoxy-d-glucose (d-GlcN3N), and d-galacturonic acid (d-GalA). Among fatty acids, three amide-linked 3-hydroxy fatty acids (12:0(3-OH) and 14:0(3-OH) and 18:0(3-OH)) were identified, whereas ester-linked fatty acids were represented by a non-polar fatty acid (18:0) and two long (ω-1)-hydroxylated fatty acids (26:0(25-OH) and 28:0(27-OH)) (Figure 1, Table 1). All fatty acids were identified based on their chromatographic properties (retention times) and characteristic mass spectra. Additionally, a short-chain organic acid (malic acid) was found in the lipid A preparation. This component has been identified based on GC-MS analysis of TMSi-derivatives of butyl esters of volatile substances liberated from *O. carboxidovorans* lipid A. Authentic malic acid was used as a standard. Obtained derivatives of both substances co-eluted and gave the same mass spectra.

### 2.2. NMR Spectroscopy of O. carboxidovorans Lipid A

The native lipid A of *O. carboxidovorans* was dissolved in chloroform-d1/methanol-d4 (6:1, *v*/*v*) and structurally characterized by 1D and 2D NMR spectroscopy. As confirmed by ^31^P NMR, the lipid A had no phosphate residues.

**Figure 2:**
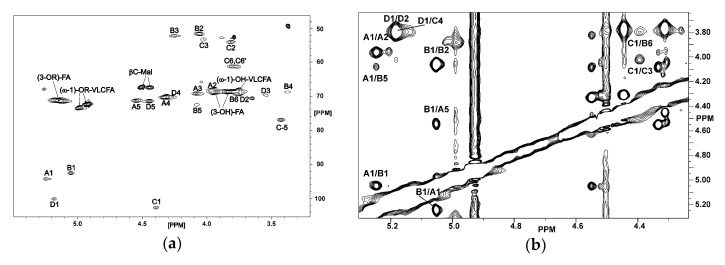
Part of the HSQC (**a**) and ^1^H-^1^H ROESY (**b**) spectra of lipid A from *O. carboxidovorans* OM5. Only signals important for structural analysis are marked.

The HSQC-DEPT spectrum (Figure 2a) contained signals from four anomeric carbons (δ_c_ 92.57–102.69), four signals of nitrogen-bearing carbons (δ_c_ 51.65–54.12), signals of remaining sugar carbons (δ_c_ 76.94–61.24), signals for CH-OH and CH-OR groups from hydroxylated fatty acids, and from the CH-OH group of malic acid (δ_c_ 67.16–73.39). Based on the ^1^H–^1^H COSY, TOCSY, and ^1^H–^13^C HMBC experiments four spin systems were identified, all deriving from hexopyranoses. Spin systems **A** and **D** derived from α-d-Gal*p*A residues, B represented α-d-Glc*p*N3N, and **C** was assigned to β-d-Glc*p*N3N. All ^1^H and ^13^C chemical shifts for the sugar backbone of *O. carboxidovorans* lipid A were assigned and listed in Table 2. The anomeric configuration of all monosaccharides was confirmed by measuring the ^1^*J*_(C1,H1)_ coupling constants. Relatively large values of coupling constants (above 170 Hz) for anomeric signals were found for residues **A**, **B**, and **D**, thus identifying their α-configuration. Residue **C** was characterized by a small (161.1 Hz) ^1^*J*_(C1,H1)_ coupling constant value, indicating its β-configuration. 

The ROESY spectrum (Figure 2b) showed the following connectivities between anomeric and linkage protons: **A1/B1** (δ 5.248/5.053), **C1/B6** (δ 4.395/3.710), and **D1/C4** (δ 5.189/3.825). These data were confirmed by analysis of the HMBC spectrum, in which the following ^1^H-^13^C connectivities were found: **A1/B1** (δ 5.248/92.57), **C1/B6** (δ 4.395/68.70), and **D1/C4** (δ 5.189/75.22). Taken together, the sugar backbone of *O. carboxidovorans* lipid A has the following structure:

**D        C        B        A**

α-d-Gal*p*A-(1→4)-β-d-Glc*p*N3N-(1→6)-α-d-Glc*p*N3N-(1→1)-α-d-Gal*p*A

The chemical shift values of the α, β, and γ carbons and protons of the 3-OH-fatty acids (both 3-O-acylated and those with a free OH group) and for signals derived from ω, ω-1, ω-2, ω-3, and ω-4 protons and carbons of substituted as well as unsubstituted (ω-1)-hydroxylated long chain fatty acids were established and shown in Table 3. These data were similar to those reported for lipid A derived from *B. elkanii* or *B. japonicum* [12,13]. The NMR signals from α and β carbons and protons as well as signals from carbons of carboxyl groups belonging to malic acid residues were identified as well. Two signals derived from β-CH proton and carbon at δ 4.505/67.16 and 4.446/67.41 were recognized. The signals derived from carbons of malic acid carboxyl groups (δ 170.84 and 173.30) showed an interresidue correlation with (ω-1)-hydrogens (δ 4.929 and 4.996, respectively) from the long chain fatty acids in the HMBC spectrum. The signals of the β-CH group of unsubstituted 3-hydroxy fatty acids were identified at δ 3.833/68.80 and 3.942/68.80. Two signals derived from the β-CH proton and carbon of 3-O-substituted fatty acids were found at δ 5.262/68.0 and 5.145/71.29. The proton/carbon chemical shifts at δ 4.996/73.39 and 4.929/72.36 were derived from the (ω-1) methine groups of the long chain fatty acids, which are connected to the 3-OH group of the amide-linked fatty acids and bear malic acid as a tertiary residue.

### 2.3. Mass Spectrometry of O. carboxidovorans Lipid A

**Figure 3:**
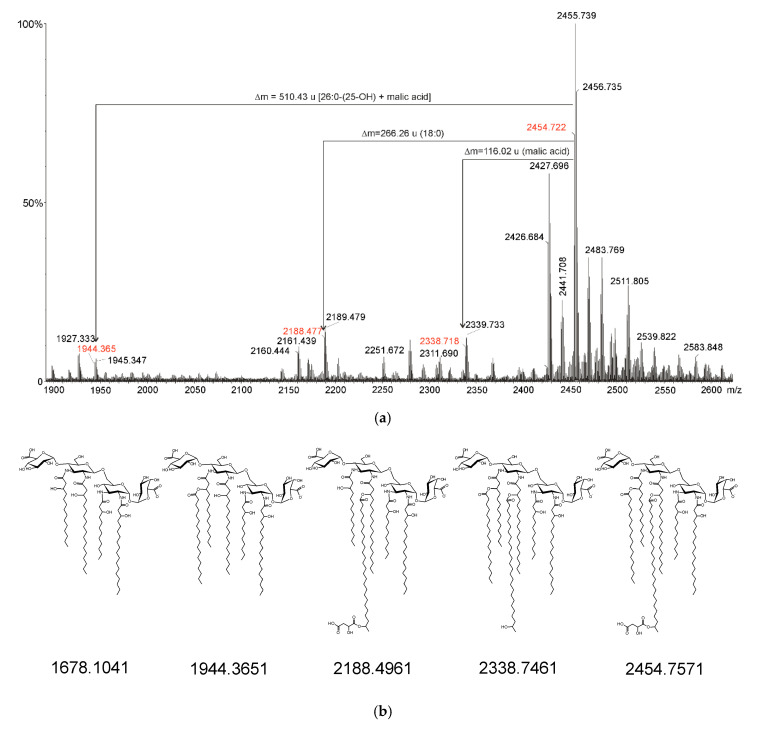
Negative mode MALDI-TOF mass spectrum of native lipid A from *O. carboxidovorans* OM5 (**a**); proposed formulas of lipid A ions presented in the lipid A mass spectrum (with calculated *m/z* values) (**b**). The *m/z* values marked in red at part (**a**) refer to the relevant chemical formulas given in part (**b**) of the figure.

Mass spectrometry experiments were performed in the positive and negative ion modes of native lipid A preparations. Figure 3 shows the negative-ion MALDI-TOF mass spectrum of the native lipid A sample.

Based on the chemical analyses of the sugar components and fatty acid residues, the most abundant signal at *m/z* 2454.722 could be assigned to lipid A molecules containing two Glc*p*N3N residues, two Gal*p*A units, 12:0(3-OH), 14:0(3-OH), two 18:0(3-OH) fatty acid residues, and two ester-linked fatty acids: 18:0 and 26:0(25-OH) acids. The last one was additionally esterified by malic acid. Around this signal, one can distinguish a number of others with a lower intensity. The mass differences between the next two (e.g., *m/z* 2441.708 and 2455.739 or 2427.696 and 2455.739) correspond to the mass of a single or double methylene groups (14 or 28 u), as a result of a different acylation pattern. Moreover, this spectrum (Figure 3) contains signals corresponding to lipid A molecules lacking an octadecanoate (*m/z* at 2188.477) or acyloxyacyl (26:0(25-OH) + malate, *m/z* at 1944.382) residue or both substituents simultaneously (*m/z* at 1678.123, traces). As a result, all of these signals should be thought to prove the existence of the whole set of particles being variants of lipid A differing in acyl substitution and acyl substituents. It should be noted, however, that most of the molecules in the preparation are lipid A molecules substituted with seven acyl residues (four 3-OH-fatty acids, one VLCFA, one non-polar acid, and malic acid). Based on the analysis of the intensity of the respective ions in the mass spectrum of native lipid A, it was estimated that ca. 85% of the molecules in the preparation were heptaacylated lipid A. Each of the negative ions described above corresponds to two ions ([M + H]^+^ and [M + Na]^+^), which could be observed in the MALDI-TOF spectrum in the positive-ion mode. Fragmentary positively charged ions were observed in this spectrum as well. The most informative of these are B^+^ type ions. An ion at *m/z* 2262.726 was identified. It can be treated as a B_3_^+^ ion. Its presence indicates substitution of the lipid A sugar backbone at the C-1 position by a galacturonic acid residue. The ion at *m/z* 1622.242 represents a B_2_^+^ charged molecule and indicates asymmetrical distribution of secondary and tertiary fatty acids in lipid A molecules. These (VLCFA, 18:0 and malic acid) decorate the distal Glc*p*N3N in lipid A molecules. Moreover, the presence of ions in the spectra (both positive and negative-ion modes) representing lipid A molecules lacking malate (*m/z* 2338.752 in negatiove-ion mode) and lacking both the VLCFA residue and the malic acid residue (*m/z* 1944.382 in negatiove-ion mode), as well as the simultaneous absence of ions corresponding to lipid A molecules lacking VLCFA but not malate clearly demonstrate that VLCFA and malic acid form an acyloxyacyl residue having the following structure 26:0[25-O-(C=O)CH_2_CH(OH)COOH].

**Figure 4:**
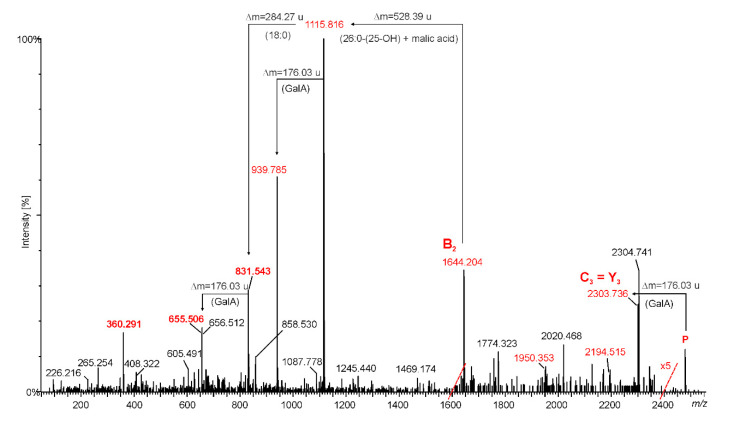
MALDI-TOF MS/MS spectrum of the parent ion at *m/z* 2478.775 (heptaacylated lipid A of *O. carboxidovorans*). Peaks between *m/z* 1600 and 2400 are magnified five times. Ions marked in red are discussed in the text.

Positive-ion mode MS/MS analysis was performed to reveal the exact positions of the lipid A acyl moieties (and in this way to prove the conclusions formulated above). The MALDI TOF MS/MS spectrum of the ion at *m/z* 2478.775 [M_heptaacyl_ + Na]^+^ is shown in Figure 4. Ion peaks originating from cleavage of the glycosidic linkage, marked as B, C, and Y (according to Domon and Costello nomenclature [19]) are not prominent and not very intense in this spectrum. Two of them exhibit slightly higher intensity and can undoubtedly be attributed to specific chemical structures; moreover, monoisotope masses calculated on the basis of the proposed formulas fits the experimental data very well. The first ion is at *m/z* 2303.736. It can be interpreted as the sodiated ion of the C_3_ type or as the sodiated ion of the Y_3_ type. Both have identical molecular masses and both arise through the elimination of Gal*p*A from lipid A (parent ion). The second ion observed at *m/z* 1644.204 is marked as B_2_^+^. This fragment ion is made up of one Gal*p*A, one Glc*p*N3N, two 3-hydroxylated fatty acids (14:0(3-OH), and 18:0(3-OH)) and 18:0 as well as 26:0(25-O-(C=O)CH_2_CH(OH)COOH) moieties. The composition of this ion complements the sodium atom. Further fragmentation of B_2_^+^ yields two predominant ions in the spectrum. Elimination of VLCFA (with its *O*-acyl substituent) leads to an ion at *m/z* 1115.816 and subsequent elimination of Gal*p*A gives an ion at *m/z* 939.785. The loss of octadecanoic acid (Δ = 284.26 u) from both ions described above gives rise to ions at *m/z* 831.543 and 655.506, respectively. The last prominent signal in the spectrum at *m/z* 360.291 corresponds to the C_21_H_39_N_1_O_2_Na formula (theoretically calculated monoisotopic mass 360.2873 u) and is presumably made up of dehydrated 18:0(3-OH) connected via an amide bond to a fragment of the distal GlcN3N including only the C1, C2, and C3 carbons and one oxygen. 

The fragmentations described above clearly indicate that two acyls (18:0 and 26:0(25-O-(malic acid)) are ester-linked, thus demonstrating that they occur as secondary fatty acids. Moreover, this observation unambiguously proved again that both secondary O-substitutions occurred on the non-reducing Glc*p*N3N unit. Therefore, *O. carboxidovorans* OM5 displayed a lipid A structure with an asymmetric distribution of the acyl moieties with respect to the di-Glc*p*N3N backbone (symmetry 4+2 or, more precisely, taking into account malic acid as a separate residue, the symmetry should be written as follows: 5+2).

**Figure 5:**
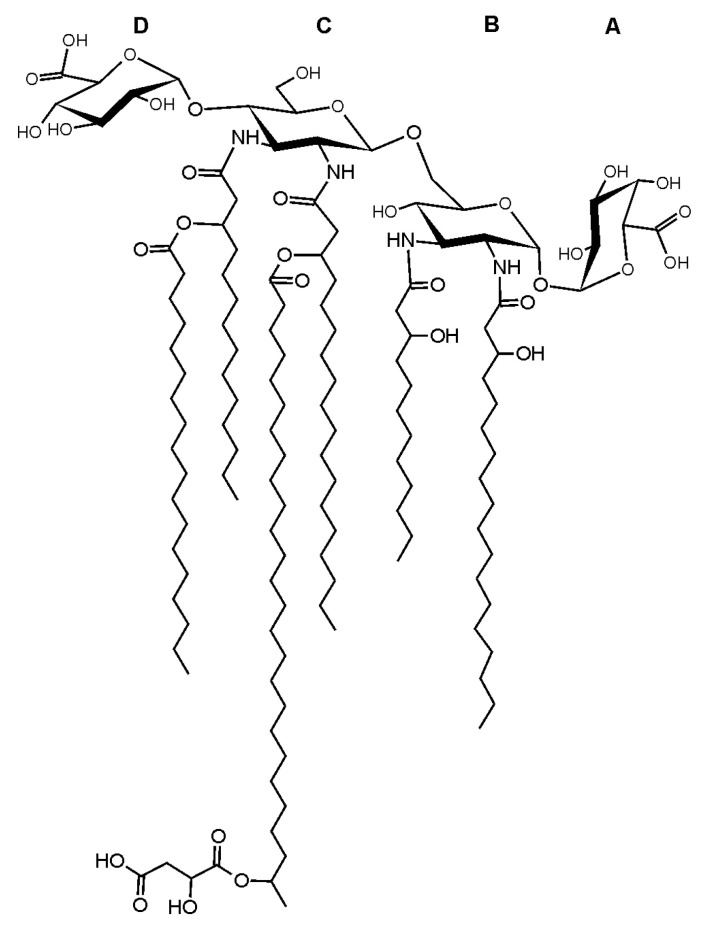
Proposed structural formula for the most complex *O. carboxidovorans* OM5 lipid A molecule. M_mi_ = 2455.76435 u.

Summing up all the experimental data, a probable structural formula of lipid A from *O. carboxidovorans* OM5 can be proposed (Figure 5).

### 2.4. Genomic Studies 

The experimental data showing the structure of *O. carboxidovorans* OM5 lipid A obtained with MALDI-TOF mass spectrometry and NMR spectroscopy were further supplemented by in silico analyses of the *O. carboxidovorans* OM5 genomic sequence aiming to identify putative genes encoding proteins/enzymes engaged in the lipid A biosynthesis pathway.

The sequence similarity of the 16S rRNA gene indicates that *O. carboxidovorans* is closely related to the members of the *Bradyrhizobiaceae* family [6]. Our results thereby confirm that the structure of the *O. carboxidovorans* lipid A sugar backbone is identical to that of *Aquifex pyrophilus* [20] and *Caulobacter crescentus* [21]. In turn, at its reducing end, it resembles some mesorhizobial lipids A (i.e., *M. huakuii* IFO 15243T and *M. loti* MAFF303099) [10,11]. Moreover, at the non-reducing end, it resembles some rhizobial lipids A [9]. The lipid A structure as well as gene clusters engaged in its biosynthesis have been recognized for *M. loti* MAFF303099 and *Rhizobium leguminosarum* 3841; therefore, respective protein sequences of these model strains were used as queries in BLAST similarity searches comprising the *O. carboxidovorans* OM5 genome sequence (Table 4). Using this approach, we recognized a set of putative genes coding for common enzymes required for the lipid A biosynthesis (i.e., *lpxA-D*, *lpxH*, *lpxK*, and *kdtA*, data for these genes are not shown) and genes encoding specific enzymes involved in the structural modifications of lipid A (*lpxE*, *lpxF*, *rgtD*, *rgtF*, *rgtE* and *acpXL-lpxXL* cluster) found in some Gram-negative bacteria (Table 4 and Table 5). Additionally, using *M. loti*, lipid A biosynthesis related proteins as queries in the genome of *O. carboxidovorans* sequences coding for putative enzymes involved in the conversion of Glc*p*N to Glc*p*N3N (presumable homologs of *gnnA* and *gnnB*) were identified (Table 5). Such enzymes, essential for the biosynthesis of the Glc*p*N3N type of the lipid A disaccharide backbone, are specific not only for *Mesorhizobium* but also for the *Azorhizobium* and *Bradyrhizobium* genera. Putative ORFs detected in the *O. carboxydovorans* OM5 genome ascribed to lipid A biosynthesis/modification shared sequence similarity with the respective proteins of *M. loti* MAFF 303099 or *R. leguminosarum* bv. Viaciae 3841 (reaching even 78% for individual proteins encoded in the *acpXL-lpxXL* cluster) (Table 4 and Table 5), strongly suggesting their engagement in this biosynthetic pathway. Due to the identical structures of the sugar part of lipid A of *O. carboxidovorans* OM5 and *Aquifex pyrophilus*, the database searches aiming to identify putative genes necessary for Glc*p*N3N-(1→6)-Glc*p*N3N backbone biosynthesis were extended to representatives of *Aquifex* and *Caulobacter*. In the *Aquifex* genus, *Aquifex aeolicus* VF5 is the only accessible fully sequenced genome (lack of genomic data for *A. pyrophilus*). In the *A. aeolicus* VF5 genome, putative homologues of the *rgtD*, *rgtF*, and *rgtE* genes were identified (Table 4). Surprisingly, we were not able to detect sequences similar to *lpxE* and *lpxF* (encoding hypothetical lipid A phosphatases) in the *A. aeolicus* VF5 genome. These enzymes were shown to be involved in dephosphorylation of the lipid A precursor during the biosynthesis of LPS in *R. leguminosarum* bv. Viciae 3841 [11]. It may be assumed that the process of phosphate residue removal from both ends of the Glc*p*N3N-disaccharide in representatives of the *Aquifex* genus is catalyzed by other types of unknown phosphatases. 

As demonstrated by previously published structural data for lipid A of *Caulobacter crescentus* CB15 [21], putative homologues of *lpxE*, *rgtD*, *rgtF*, and *rgtE* were found in its genome (Table 4). In the reference genomes, the *rgtDFE* genes encode hypothetical 4′-α-Gal*p*A, α-(1→1)-Gal*p*A, and bactoprenyl-phosphate-Gal*p*A transferases, respectively [11]. The *C. crescentus* CB15 ORF marked as CC_3019 displayed some sequence similarity with putative lipid A 1-phosphatase encoded by *lpxE* in *R. leguminosarum* bv. Viciae 3841 [22]. However, using this approach, no putative homologues of lipid A 4’-phosphatase were found in the *C. crescentus* CB15 genome. In *Aquifex*, *Azorhizobium* (data not shown), *Mesorhizobium*, and *Oligotropha* homologues of *rtgE* and *rtgF* are clustered; however, it seems not to be a prerequisite for respective protein products to participate effectively in lipid A modification (see *R. leguminosarum*, Table 4). On the other hand, the available data strongly suggest that genes responsible for the synthesis and incorporation of VLCFAs into lipid A always form a tight cluster [9]. Although a set of genes presumably engaged in VLCFA production was found in *C. crescentus* CB15 (Table 5), they were dispersed over the bacterial genome, and the formation and incorporation of VLCFAs was not observed in these bacteria.

## 3. Discussion

In this work, we have described the structure of *O. carboxidovorans* OM5 lipid A, which contains a β-d-Glc*p*N3N-(1→6)-α-d-Glc*p*N3N disaccharide decorated on both sides (at positions C-1 and C-4’) by α-d-Gal*p*A residues. This extended sugar backbone is unique in bacteria. To date, there have been only two reports of the existence of similar lipids A. They are synthesized by bacteria belonging to the hypertermophilic *Aquifex pyrophilus* species [20], and the stalk-forming *Caulobacter crescentus* [21]. The substitution of only the reducing end of lipid A by α-(1→1)-d-Gal*p*A is also not common among bacteria and has been identified in lipids A from a few representative genera. These include the associative diazotroph *Azospirillum lipoferum* [23], and some rhizobia: *M. huakuii* [10], *M. loti* [11], and *P. trifolii* [24]. On the other hand, the substitution of exclusively the non-reducing end of lipid A by α-d-Gal*p*A-(1→ at position C-4’ is not common either, and has been identified in lipids A from a few representatives of rhizobia (*R. etli* CE3, *R. leguminosarum* bvs. Trifolii and Viciae) [9].

The amino groups of both Glc*p*N3N of *O. carboxidovorans* OM5 lipid A are symmetrically substituted by 3-hydroxyoctadecanoic fatty acid at position C-2 and C-2’. In the dominant type of lipid A molecules, the amino group at position C-3 is acylated with 3-hydroxylauric acid, while a 3-hydroxymyristoyl group is located at position C-3’. Due to the multiplicity of signals assigned to heptaacylated lipid A, another distribution of primary fatty acids in individual subgroups of lipid A molecules should also be expected. Even if the extended backbones of lipids A synthesized by *Oligotropha*, *Aquifex*, and *Caulobacter* are the same, the substituting primary fatty acids are significantly different. Lipid A contains mainly 18:0(3-OH) (or 14:0(3-OH) and 12:0(3-OH)):residues in *Oligotropha*, 16:0(3-OH) (and 14:0(3-OH)) residues in *Aquifex*, and almost exclusively 12:0(3-OH) residues in *Caulobacter*. This fact can be explained by the different basic metabolisms of fatty acids of these bacteria [7,25,26].

The 28:0(27-OH) fatty acid is the most often isolated VLCFA-type fatty acid among rhizobia [27], but the 26:0(25-OH) fatty acid took its place in *Oligotropha*. In addition, 25-hydroxyhexacosanoic acid was almost totally acylated with a malic acid residue. It should be emphasized that this type of tertiary substituent has not been described in the literature so far. Since ester-linked fatty acids (18:0 and 26:0(25-OH) are secondary substituents of the distal Glc*p*N3N, the complete lipid A is asymmetrically acylated and resembles the *E. coli* lipid A pattern denoted by formula 4+2 (or more precisely 5 + 2) [28].

We have found structural similarities in the acylation of lipid A with VLCFA between *O. carboxidovorans* and some rhizobia. Moreover, we have shown that the sugar backbone of lipid A in *O. carboxidovorans* is the same as in *A. pyrophilus* and *C. crescentus*. Additionally, structural similarities among the sugar backbones of lipid A of *O. carboxidovorans*, *M. loti*, *R. leguminosarum*, and *P. trifolii*, were shown. These observations were confirmed at the genomic level. Putative ORFs predicted for the lipid A biosynthesis pathway in *Oligotropha* shared sequence similarity with corresponding proteins of reference microorganisms. Further genetic and biochemical studies are necessary to elucidate which putative enzymes and at which stage of biosynthesis are involved in the esterification of the hydroxyl group of VLCFA by malic acid. This issue (on tertiary acyls) remains unexplored for all rhizobial lipids A.

## 4. Materials and Methods 

### 4.1. Bacterial Strain and Culture Condition

*Oligotropha carboxidovorans* strain OM5 (type strain, DSM 1227; previously known as *Pseudomonas carboxydovorans* OM5) was obtained from the DSMZ culture collection (Leibniz-Institute, Braunschweig, Germany). The bacteria were cultivated in aerobic conditions, in a CMO (carbon monoxide oxidizer) medium containing 0.3% sodium acetate as a carbon source, at 30 °C, with aeration by vigorous shaking.

### 4.2. LPS and Lipid A Isolation and Purification

LPS was isolated from previously delipidated and enzymatically digested cells, using the hot 45% phenol/water extraction method [17,18], as described by Komaniecka and co-workers [29]. The LPS was obtained after ultracentrifugation (104,000× *g*, 4 h, 4 °C). It was found mainly in the water phase (90%). The lipid A was liberated from the LPS by mild acid hydrolysis of 210 mg of LPS using 1% acetic acid (3 h, 100 °C). After cooling in an ice bath, the hydrolysate was converted to the two-phase Bligh–Dyer system containing chloroform/methanol/water (2:2:1.8, *v*/*v*/*v*), and the resulting water and organic phases were separated by centrifugation (4000× *g*, 15 min, 20 °C) [30,31]. The organic phase containing a lipid A portion was collected, washed twice with a water phase from the freshly prepared two-phase Bligh–Dyer system, and evaporated to dryness. Crude lipid A was stored at −20 °C as a solution in chloroform/methanol (3:1, *v*/*v*).

### 4.3. Sugar and Fatty Acid Analysis

The sugar composition of lipid A was established by hydrolysis with 2 M trifluoroacetic acid (100 °C, 4 h) and conversion of liberated monosaccharides into (amino)alditol acetates [32]. Fatty acids were analyzed after acid hydrolysis (4 M HCl_aq_., 100 °C, 4 h), extraction with chloroform (1:1, *v*/*v*), and methanolysis (0.5 M HCl/methanol, 85 °C, 2 h). Liberated hydroxy fatty acids were converted to trimethylsilyl (TMSi) derivatives of their methyl esters. Fatty acids and volatile components of lipid A obtained after methanolysis (2 M HCl/methanol, 85 °C, 18 h) were converted to butyl esters by weak butanolysis (1 M HCl/butanol, 65 °C, 2 h) and TMSi derivatized. All preparations were analyzed by GC-MS using the Agilent Technologies GC System 7890A connected to a mass selective detector (inert XL EI/CI MSD 5975C) equipped with a HP-5MS column (30 m × 0.25 mm). Helium was a carrier gas with a flow rate of 1 mL min^−1^. The temperature program for sugar analysis was as follows: 150 °C for 5 min, raised to 310 °C at 5 °C·min^−1^, and the final temperature was kept for 10 min. For analysis of fatty acid derivatives, the ramp of the temperature gradient was established at 10 °C·min^−1^. The temperature program for volatile components was as follows: 50 °C for 5 min, raised to 310 °C at 5 °C·min^−1^, and the final temperature was kept for 5 min.

### 4.4. NMR Spectroscopy

For the NMR analysis, the native lipid A was dissolved in the mixture of chloroform-d1/methanol-d4 (6:1, *v*/*v*). 1D and 2D NMR spectra (homonuclear: ^1^H,^1^H COSY, TOCSY, ROESY; and heteronuclear: ^1^H,^13^C HSQC, HSQC-TOCSY, and HMBC) were recorded using a Bruker Avance III 700 MHz spectrometer at 25 °C. Data were analyzed using TopSpin 3.2 (Bruker) software. Proton and carbon chemical shifts were measured in relation to the TMS as an internal standard (δ_H_ 0.00, δ_C_ 0.0).

### 4.5. Mass Spectrometry

MALDI-TOF mass spectrometry was performed with a SYNAPT G2-S*i* HDMS instrument (Waters Corporation, Milford, MA, USA) equipped with a 1 KHz Nd:YAG laser system (355 nm wavelength). Acquisition of the data was performed using MassLynx software version 4.1 SCN916 (Waters Corporation, Wilmslow, UK). Mass spectra were assigned with a multi-point external calibration using red phosphorous (Sigma). The lipid A samples were dissolved in chloroform/methanol (3:1, *v*/*v*) at a concentration of 10 µg/µL, and 2 mM of EDTA was added. A sample (1 µL) was transferred into the target plate wells covered with a thin matrix film. The matrix solution was prepared from 2’,4’,6’-trihydroxyacetophenone (THAP) (200 mg/ml in methanol) and mixed with nitrocellulose (NC) (15 mg/ml, suspended in 2-propanol/acetone (1:1, *v*/*v*)) in a proportion of 4:1 (*v*/*v*), as previously described by Silipo and co-workers [33]. Spectra were recorded in positive and negative ion modes. 

### 4.6. Bioinformatics Analysis

Standard BLAST setting (with the cut-off E-value of 10^−5^) was used in searching for putative proteins engaged in the biosynthetic pathway of *Oligotropha* lipid A. Protein sequences of *M. loti* MAFF 303099 and *R. leguminosarum* 3841 were used as queries in the TBLASTN searches against a genomic sequence of the *O. carboxidovorans* OM5 strain registered in the NCBI online Database. Individual BLAST protein subjects were then compared across their entire span with reference sequences using an online Global Alignment tool (with the Needleman–Wunsch algorithm) provided by the National Center for Biotechnology Information (NCBI).

## Figures and Tables

**Table 1:** Fatty acid components of *O. carboxidovorans* OM5 lipid A.

Component	Amount [µg/mg Lipid A]
12:0(3-OH)	39.7
14:0(3-OH)	24.5
18:0(3-OH)	95.7
16:0	5.7
18:0	93.7
18:1	4.6
22:0	33.2
24:0	6.5
26:0(25-OH)	112.5
28:0(27-OH)	18.2

**Table 2:** ^1^H and ^13^C NMR chemical shifts [δ; ppm] of the *O. carboxidovorans* OM5 lipid A backbone.

Spin System	^1^*J*_(C-1,H-1)_ [Hz]	H-1 C-1	H-2 C-2	H-3 C-3	H-4 C-4	H-5 C-5	H-6; H-6’ C-6
**A**, α-d-Gal*p*A-(1→	173.8	5.248	3.96	4.08	4.333	4.55	-
		94.24	68.62	69.13	70.34	71.3	171.5
**B**, →6)-α-d-Glc*p*N3N-(1→	171.8	5.053	4.059	4.255	3.375	4.078	3.710; 3.710
		92.57	51.65	52.23	68.73	72.48	68.7
**C**, →4)-β-d-Glc*p*N3N-(1→	161.1	4.395	3.816	4.027	3.825	3.427	3.773; 3.812
		102.69	54.1	53.41	75.22	76.94	61.24
**D**, α-d-Gal*p*A-(1→	170.6	5.189	3.796	3.773	4.316	4.446	-
		100.02	68.68	68.74	70.15	71.46	170.5

**Table 3:** ^1^H and ^13^C NMR chemical shifts [ppm] of acyl substituents of *O. carboxidovorans* OM5 lipid A. Underling means assigning the signal to the specific group.

Fatty Acid	Group	^1^H	^13^C
Olefinic protons/carbons (double bond)	-CH_2_-HC=CH-	5.350	130.08
	-CH_2_-HC=CH-	2.016	27.49
I^st^ –(3-OR’)-FA	α_1_/α_2_	2.620/2.504	41
	β	5.145	71.29
	γ	1.588	34.4
	R-CONH-Sug		171.51
	R-COO-		174.53
	ω	1.59	19.8
II^nd^ –(3-OR”)-FA	α_1_/α_2_	2.614/2.401	40.9
	β	5.262	68
	γ	ND	ND
	R-CONH-Sug		169.72
	R-COO-		ND
I^st^ –[(ω-1)-OR]-VLCFA	ω	1.222	19.8
	ω-1	4.996	73.39
	ω-2	1.600/1.486	36
	ω-3	1.41	25.5
	ω-4 and next CH_2_ groups	1.59	29.8
	R-(COO-) from malic acid		173.3
II^nd^ –[(ω-1)-OR]-VLCFA	ω	1.222	19.8
	ω-1	4.929	73.36
	ω-2	1.600/1.488	36
	ω-3	1.474	25.5
	ω-4 and next CH_2_ groups	1.59	29.8
	R-(COO-) from malic acid		170.84
I^st^ –(3-OH)-FA with unsubstituted OH group	α_1_/α_2_	2.339/2.252	43.9
	β	3.942	68.8
	γ	1.435	37.5
	δ	1.399	25.8
	CH_2_ groups from acyl chain	1.266	29.9
II^nd^ – (3-OH)-FA with unsubstituted OH group	α_1_/α_2_	2.307/2.201	43.05
	β	3.833	68.8
	γ	1.435	37.6
	δ	1.399	25.7
	CH_2_ groups from acyl chain	1.266	29.9
[(ω-1)-OH] VLCFA with unsubstituted OH group	ω-1	3.756	68.4
	ω-2	1.387/1.478	39.34
	ω-3	1.28	26.05
	ω-4 and next CH_2_ groups	1.59	29.94
I^st^ Malic acid	α_1_/α_2_	2.860/2.717	39.41
	β	4.505	67.16
	COO-		170.84
	R-COO-		175.36
II^nd^ Malic acid	α_1_/α_2_	2.860/2.717	39.24
	β	4.446	67.41
	COO-		170.58
	R-COO-		173.3

ND—not detected.

**Table 4:** Sequence similarity of putative proteins involved in lipid A modification in *Oligotropha carboxidovorans* OM5, *Aquifex aeolicus* and *Caulobacter crescentus* CB15 with enzymes of known functions from the reference strains: *Rhizobium leguminosarum* bv. Viciae 3841 and *Mesorhizobium loti* MAFF303099).

Gene Name (Putative Protein Function)	*lpxE* (Lipid A 1-Phosphatase)	*lpxF* (Lipid A 4’-Phosphatase)	*rgtD* (4′-GalA Transferase)	*rgtF* (α-(1,1)-GalA Transferase)	*rgtE* (Bactoprenyl/Phosphate GalA Transferase)
Reference sequence of: *R. leguminosarum* 3841 and/or *M. loti* MAFF303099	RL_RS24225 (*RL4708*)	RL_RS08140 (*RL1570*)	RL_RS03600 (*RL0684*)	MAFF_RS01135 ^2^ (*mlr0011*)	RL_RS07630 (*RL1470*)
*O. carboxidovorans* OM5 % Identity ^1^	OCA5_c19740 31.97	OCA5_cRS09935 30.96	OCA5_RS09935 28.81	OCA5_c19750 39.28	OCA5_c19730 34.69
*Aquifex aeolicus* VF5 % Identity ^1^	ND	ND	aq_765 29.31	aq_1695a 22.73	aq_1899 39.82
*Caulobacter crescentus* (*vibroides*) CB15 % Identity ^1^	CC_3019 29.84	ND	CC_0209 31.66	CC_0468 33.27	CC_0469 33.33
Putative function ascribed on BLAST sequence similarity	**phosphatase PAP2 family protein**	**phosphoesterase PA-phosphatase related**	**dolichyl-phosphate-mannose-protein mannosyltransferase**	**glycosyl transferase**	**glycosyl transferase, family 2**

^1^ % Identity with reference sequence. ^2^ *R. leguminosarum* bv. Viciae 3841 synthetize lipid A without GalA connected to proximal GlcN [9] and gene coding for α-(1,1)-GalA transferase is not present in its genome. Therefore, respective coding sequence mlr0011 from *M. loti* MAFF303099 [11] was used as reference in similarity searches.

**Table 5:** Sequence similarity of putative proteins involved in lipid A modification in *Oligotropha carboxidovorans* OM5 and *Caulobacter crescentus* CB15 with enzymes of known functions from the reference strains: *Mesorhizobium loti* MAFF303099 and *Sinorhizobium meliloti* 1021.

Gene Name (Putative Protein Function)	*gnnA* (NAD-Dependent Oxidation of GlcN 3-OH of UDP-GlcNAc)	*gnnB* (Transamination to Form UDP-GlcNAc3N)	*acpXL* (Acyl Carrier Protein)	*fabZXL* (3R)-Hydroxyacyl-ACP Dehydratase	*fabF2XL* (Beta-Ketoacyl-ACP Synthase)	*fabF1XL* (Beta-Ketoacyl-ACP Synthase)	*adh2XL* (Zinc-Binding Dehydrogenase)	*lpxXL (msbB)* (VLCFA Acyltransferase to C2’ Position of Lipid A)
Reference sequence of *M. loti* MAFF303099 and/or *S. meliloti* 1021 (old description)	MAFF_RS12135 (*mll2825I*)	MAFF_RS30880 (*mll7579*)	MAFF_RS05885 (*mlr1174*)	SM_RS09960 (*SMc04277*) ^2^	MAFF_RS05890 (*mlr1176*)	MAFF_RS05895 (*mlr1177*)	MAFF_RS05900 (*mlr1178*)	MAFF_RS05905 (*mlr1179*)
*Oligotropha carboxydovorans* OM5 (DSM1227) % Identity ^1^	OCA5_c26370 32.37	OCA5_c26360 55.41	*acpXL* 78.49	*fabA* 50.34	OCA5_c15570 43.59	*fabF1* 58.12	OCA5_c15550 62.24	OCA5_c15540 38.36
*Caulobacter crescentus* CB15 % Identity ^1^	CC_1225 25.29	CC_3611 53.12	CC_1677 39.29	CCNA_01989 36.59	CC_1678 23.76	CC_1678 31.54	CC_1569 30.41	CC_2250 32.26
						CC-3719 30		
Putative function ascribed on BLAST sequence similarity	oxidoreductase domain protein	DegT/DnrJ/EryC1/StrS aminotransferase	acyl carrier protein AcpXL	beta-hydroxyacylACP dehydratase	beta-ketoacyl synthase	3-oxoacyl-[ACP] synthase 2	alcohol dehydrogenase	VLCFA acyltransferase to C2’ position of lipid A

^1^ % Identity with reference sequence. ^2^ *M. loti* MAFF303099 synthetize VLCFAs using housekeeping FabZ and region in *M. loti* genome encoding enzymes responsible for VLCFAs biosynthesis does not contain fabZXL homolog. Therefore, respective coding sequence SMc04277 from *S. meliloti* 1021 was used as reference in similarity searches [9].

## Abbreviations

EDTA	ethylenediaminetetraacetic acid
Glc*p*N3N	2,3-diamino-2,3-dideoxyglucose
Gal*p*A	galacturonic acid
MALDI-TOF	matrix assisted laser desorption/ionization—time of flight
NC	nitrocellulose
std	internal standard
Sug	sugar residue
THAP	2′,4′,6′-trihydroxyacetophenone
TMSi	trimethylsilylating reagent
VLCFA	very long chain fatty acid
